# Angiocrine Regulation of Epithelial Barrier Integrity in Inflammatory Bowel Disease

**DOI:** 10.3389/fmed.2021.643607

**Published:** 2021-08-02

**Authors:** Michael Stürzl, Meik Kunz, Susanne M. Krug, Elisabeth Naschberger

**Affiliations:** ^1^Division of Molecular and Experimental Surgery, Department of Surgery, Universitätsklinikum Erlangen, Friedrich-Alexander University (FAU) of Erlangen-Nürnberg, Erlangen, Germany; ^2^Chair of Medical Informatics, Friedrich-Alexander-University (FAU) of Erlangen-Nürnberg, Erlangen, and Fraunhofer Institute of Toxicology and Experimental Medicine, Hannover, Germany; ^3^Clinical Physiology/Nutritional Medicine, Charité-Universitätsmedizin Berlin, Berlin, Germany

**Keywords:** endothelial, angiocrine, barrier, inflammatory bowel disease, inflammation, angiogenesis, epithelial

## Abstract

Inflammatory bowel disease describes chronic inflammatory disorders. The incidence of the disease is rising. A major step in disease development is the breakdown of the epithelial cell barrier. Numerous blood vessels are directly located underneath this barrier. Diseased tissues are heavily vascularized and blood vessels significantly contribute to disease progression. The gut-vascular barrier (GVB) is an additional barrier controlling the entry of substances into the portal circulation and to the liver after passing the first epithelial barrier. The presence of the GVB rises the question, whether the vascular and endothelial barriers may communicate bi-directionally in the regulation of selective barrier permeability. Communication from epithelial to endothelial cells is well-accepted. In contrast, little is known on the respective backwards communication. Only recently, perfusion-independent angiocrine functions of endothelial cells were recognized in a way that endothelial cells release specific soluble factors that may directly act on the epithelial barrier. This review discusses the putative involvement of angiocrine inter-barrier communication in the pathogenesis of IBD.

## Clinical Presentation and Epidemiology of Inflammatory Bowel Disease

Inflammatory bowel disease (IBD) includes inflammatory diseases of the colon and small intestine with Crohn's disease and ulcerative colitis being the major clinical presentations (1). Crohn's disease affects the small intestine and large intestine, as well as the mouth, esophagus, stomach and the anus, whereas ulcerative colitis primarily affects the colon and the rectum (2). Crohn's disease and ulcerative colitis are different diseases, but commonly present with any of the following symptoms: abdominal pain, diarrhea, rectal bleeding, severe internal cramps/muscle spasms in the region of the pelvis and weight loss. In addition, anemia is a common extra-intestinal complication of IBD.

IBD is classically considered as a disease of Westernized countries but has started to rise worldwide in the beginning of the twenty first century (3). The rise is population-dependent and categorized into four different epidemiological stages: first, the *Emergence Stage* with sporadic cases of IBD observed in developing countries, second, the *Acceleration in Incidence Stage* with rising incidence and relatively low prevalence in newly industrialized countries, third, the *Compounding Prevalence Stage* with stable incidence and steeply rising prevalence in countries of the Western world, and forth, the *Prevalence Equilibrium Stage*, which represents the opposing forces between an aging IBD population and the incidence of IBD. In Germany at present 620,085 persons are suffering from IBD with a predicted rise up to 815,200 patients in 2030. In the U.S. presently 2,489,362 patients are registered and a rise up to 3,544,480 is expected within the next 10 years (4).

IBD is characterized by a chronically relapsing intestinal inflammation that is thought to result from an exaggerated immune response to the commensal microbiota. However, the specific molecular mechanisms driving IBD pathogenesis are still unclear. Many different putative susceptibility genes for IBD are reported but all of these are associated with only low risk and differ in different countries of the world. At present, it is commonly accepted, that cytokines, such as, tumor necrosis factor (TNF), interleukin (IL)-10, transforming growth factor (TGF)-β, IL-6, IL-12, IL-13, IL-17, IL-21, IL-23, interferon (IFN)-γ and C-X-C motif chemokine ligand (CXCL)10, are drivers of the excessive immune response, leading to leukocyte infiltration and mucosal damage. In addition, there is agreement that IBD pathogenesis is closely associated with a loss of intestinal epithelial barrier functions associated with bacterial translocation, likely representing an initiating or early event in the disease (5–10).

Recently, it became evident that the intestinal barrier involves two sequential physical barriers. The first being the epithelial barrier consisting of a single cell layer of epithelial cells and a mucus layer which physically separates the microbiota in the gut lumen and epithelial cells (11). Directly below the epithelial barrier an additional barrier was identified, the gut-vascular barrier (GVB) controlling the entry of substances into the portal circulation and their access to the liver after passage of the first epithelial barrier (12, 13). The discriminative control of nutrient uptake and tight sealing towards potentially pathological microorganisms requires a profound regulation of the barrier permeability.

## Structure and Function of the Epithelial Barrier in IBD

The epithelial barrier allows the co-existence of commensal microbiota and mucosal immune cells in the gut. It consists of a physical barrier established by the epithelial cells situated on a basement membrane. Collagen type IV and laminins are the predominant components of the basement membrane (14). The basement membrane is subject of continuous remodeling. Increased remodeling was observed under inflammatory conditions in association with decreased barrier functions (14). At the cellular level barrier functions are established by (i) densely packed microvilli on the apical side of intestinal epithelial cells termed the brush border (15), (ii) tight cell-cell interactions between the epithelial cells, (iii) the cellular resistance to bacterial transcytosis (16), and (iv) specialized epithelial cells, such as mucus-producing goblet cells and anti-microbial peptide secreting Paneth cells (12). Altogether, the epithelium exerts manifold functions, establishing a physical barrier against pathogen invasion and also performing innate immune functions and nutrient uptake (17). Thereby, the preservation of the epithelial integrity is a major aspect in order to preserve homeostasis and to avoid the progress of inflammation in mucosal tissues (18) [for review see: Lopez-Posadas et al. (11)].

At the molecular level the intercellular barrier of the intestinal epithelium is established by apical junction complexes comprised of tight and adherens junctions. Adherens junctions consist of cadherins and nectins and are mainly important for the cell-cell-adhesion (19, 20). Tight junctions are multiprotein-complexes consisting of several transmembrane proteins: tight junction associated MARVEL proteins (TAMP) like occludin, marvelD3 and tricellulin, junctional adhesion molecules (JAM), angulins and the family of claudins, which has in mammalia 27 members that either possess barrier- or channel-forming properties affecting the overall permeability characteristics of the epithelia [for review see Günzel and Fromm (21)]. Adherens junctions as well as tight junctions establish zipper-like structures, sealing the paracellular space within the epithelial cell layer (22). These intercellular junctions are connected to the actin cytoskeleton via cytoplasmic adaptors, such as zonula occludens proteins, and catenins supporting the mechanical strength of the junctions (23–25). Cell activation with molecules that induce permeability causes actin reorganization into stress fibers. This is associated with increasing traction forces, which lead to the detachment of adherens junctions from the cytoskeleton followed by the formation of gaps between adjacent cells (26, 27). Further mechanisms such as the removal of cell-cell interaction molecules from the cell surface by internalization and/or by proteolytic cleavage can regulate the intestinal barrier permeability (11, 28).

The epithelium is constantly renewed without an effect on its tightness. Within this process stem cells at the crypt bottom proliferate and differentiate into the different intestinal epithelial cell subtypes with specialized biological functions (29). Subsequently, most of the differentiated epithelial cells migrate upwards to the villus tip, where aged cells die and are shed into the lumen (30, 31). The tightness of the epithelial layer is maintained by the intercellular junctions during this process (23). During cell shedding, epithelial integrity is maintained in cytoskeleton and membrane trafficking-dependent processes regulating the redistribution of junctional proteins along lateral membranes (32, 33).

Increased epithelial tight junction permeability is a hallmark in the gut of IBD patients (34–38). It is believed that the disruption of intercellular junctions and cytoskeleton rearrangements in the context of infection or inflammation lead to a breakdown of epithelial integrity (39–41). Although a correlation between epithelial barrier permeability and disease activity has been observed in patients with Crohn's disease, the cause of this barrier collapse is still a matter of controversy (42, 43). Experimental animal studies demonstrated that a deficiency of single tight junction proteins is not sufficient to cause pathology due to compensatory mechanisms (44, 45) with the exception of claudin-15 (46). However, agreement exists that inflammation-derived soluble mediators such as IL-6 (47), IL-13 (48, 49), TNF (50), and IFN-γ (51, 52) affect tight junctions and may increase intestinal permeability in experimental colitis models and IBD (53–55). These observations suggested that the epithelial barrier breakdown occurs as a consequence of proinflammatory cytokine stimulation. In contrast, recent studies in IBD patients demonstrated that an increase of epithelial permeability precedes flares of inflammatory bowel pointing towards a causative role of epithelial barrier breakdown in the development of intestinal inflammation (35, 56–58). The latter is supported by reports that a decrease of epithelial permeability by application of vitamin D (59, 60), probiotics (61–63), IL-22-triggered mucus production (64), butyrate (65, 66), or an anti-TNF antibody caused clinical amelioration of chronic colitis (67, 68). Moreover, alternative portals for gut leakiness such as brush border functions and intestinal bacterial endocytosis by epithelial cells have to be considered and may play important pathogenic roles providing putative targets for therapy of inflammatory bowel disease (15). Altogether, these results suggest that the epithelial barrier function is important and its maintenance can counteract the development of inflammatory bowel disease.

## The Impact of Blood Vessels on IBD Pathogenesis

**Figure 1:**
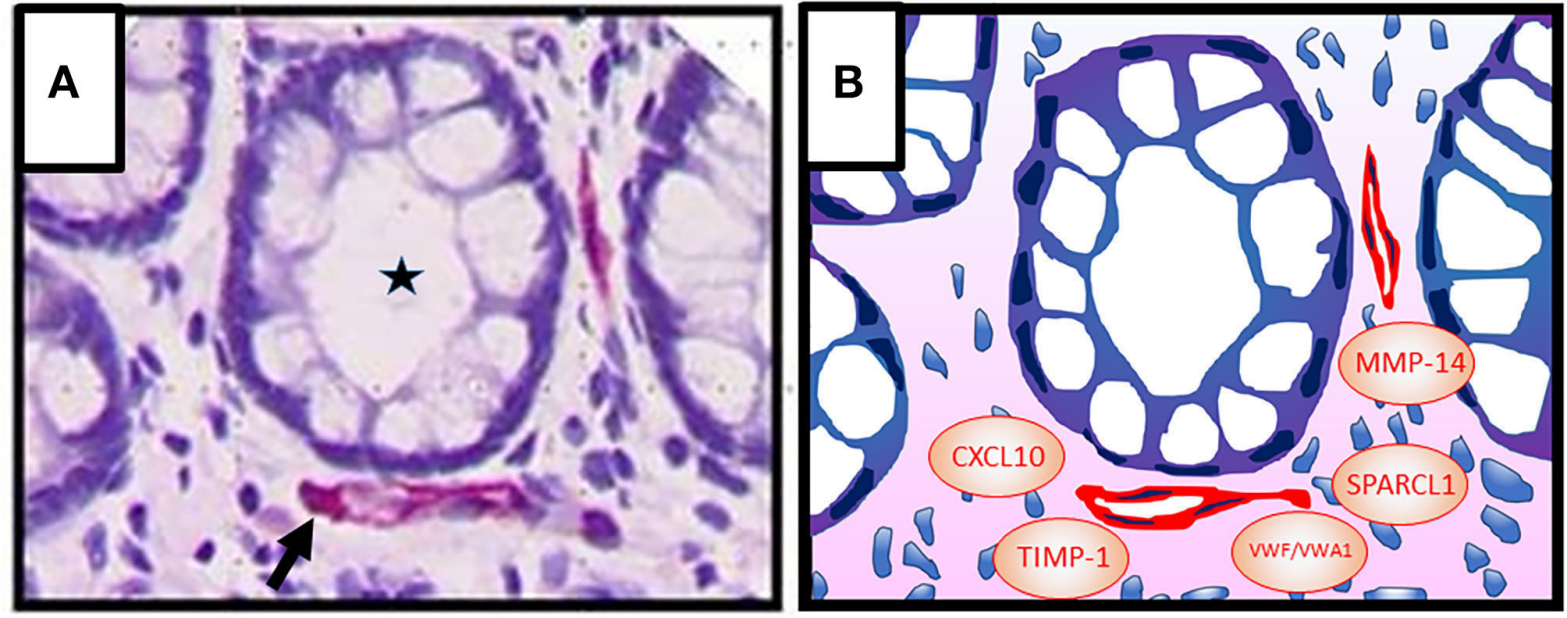
**(A)** Colonic crypt (intestinal gland, asterisk) with vessels (red, arrow) in the lamina propria. Epithelial cells (1st barrier) and endothelial cells (2nd barrier) are directly adjacent, indicating active inter-barrier communication. Vascular endothelial cells were stained immunohistochemically using an anti-CD31 antibody. Cell nuclei (blue) were stained by haematoxylin. **(B)** Graphic presentation of **(A)** indicating possible factors that may be involved in angiocrine regulation of epithelial barrier functions in IBD [von Willebrand factor A domain containing 1 (VWA1), von Willebrand factor (VWF), matrix metalloproteinase (MMP)-14, tissue inhibitor of metalloproteinases (TIMP)-1, C-X-C motif chemokine ligand (CXCL) 10, secreted protein, acidic and rich in cysteine–like 1 (SPARCL1)].

Capillaries are located in close proximity to the intestinal epithelial cell barrier (Figure 1A). Blood vessels in adult tissues evolve through sprouting from preexisting vessels, a process termed angiogenesis (69). Angiogenic activity correlates with disease severity in IBD suggesting that blood vessels may contribute to pathogenesis (70–73). Moreover, elevated levels of angiogenic growth factors including vascular endothelial growth factor (VEGF)-A and basic fibroblast growth factor (bFGF), that synergize in angiogenesis activation, have been detected in the inflamed mucosa and in the blood during active IBD (74, 75). However, experimental colitis models provided conflicting results on the contribution of angiogenesis to disease activity. Neutralization of VEGF-A resulted in a decreased vessel density and improvement of the disease in dextran sulfate sodium (DSS)–induced and 2,4,6-trinitrobenzenesulfonic acid (TNBS)–induced colitis (73, 76). In contrast, reduced angiogenic activity induced by deficiency of placental growth factor failed to ameliorate colitis in the same experimental models (77). These results indicated that besides vessel density additional parameters such as vessel quality are of relevance in IBD pathogenesis. In fact, newly formed vessels in IBD tissues are strongly disorganized and leaky as evident by associated edema (78).

The difficulties in determining the precise role of blood vessel function in IBD may be due to the fact that the intestinal endothelial cells are both, targets and regulators of inflammation (78). In this framework, IBD-associated inflammatory cytokines such as TNF-α, IL-1β and IFN-γ can activate endothelial cells by inducing the expression of adhesion molecules for leukocytes such as E-selectin, intercellular adhesion molecule (ICAM)-1 or vascular cell adhesion molecule (VCAM)-1 (79). Macrophages are important drivers of IBD and are characteristically expressing high amounts of TNF-α and IL-1β, which may amplify the extravasation of these cells being responsible for the high numbers of macrophages present in IBD tissues (80). In addition, inflammation is associated with increased angiogenesis supporting immune cell recruitment by increase of blood flow and endothelial surface (81). As mentioned above the intestinal endothelium also establishes an additional barrier in the gut, the GVB (12, 13). The GVB constitutes a semipermeable barrier between the blood stream and the interstitium regulating the transport of nutrients, tissue fluid homeostasis and the transmigration of immune cells but is non-permissive to bacterial penetration (13, 28, 78, 82). The latter is in agreement with the observation that bacterial lipopolysaccharides (LPS) in low concentrations are stabilizing the vascular barrier (83). In contrast, high concentrations of LPS (>10 μg/ml) inhibit endothelial cell migration, down-regulate intercellular junction molecules and increase the permeability of the vascular barrier (83). Paracellular (i.e., in between the cells) or transcellular (i.e., across the cells) routes are available to cross the endothelial cell monolayer. Transcellular exchange is accomplished via either solute transporters, or transcytosis via vesicular carriers (e.g., caveolae), or pore-like subcellular structures (i.e., fenestrae and transendothelial channels) (84, 85). The paracellular route is controlled by adherens junctions and tight junction proteins similar as in the epithelial barrier. In intestinal endothelial cells, tight junctions are composed mainly of occludin, junctional adhesion molecule (JAM)-A, zonula occludens (ZO)-1, and cingulin (13). Claudin-3, -5, and -12 from the claudin family are known to be mainly expressed in endothelia (86, 87).

Adherens junctions are formed by vascular endothelial (VE)-cadherin and β-catenin (13). Of note, the same cytokines regulating immune cell extravasation can also deregulate adherens and tight junction formation in endothelial cells supporting translocation of bacteria thereby further amplifying the inflammatory process [for review see: Lopez-Posadas et al. (11)].

The impact of the GVB in intestinal inflammation is substantiated by mouse models of acute and chronic DSS-colitis. In these models intestinal vessel perfusion remained constant during colitis whereas vessel permeability strongly increased (5). Using experimental animal models with an endothelial cell specific knockout of the interferon-γ-receptor 2 (IFNγR2) it was shown that the IBD-associated cytokine IFN-γ induces a breakdown of the vascular barrier based on the disruption of the adherens junction protein VE-cadherin and this was significantly increasing DSS-induced experimental colitis. Importantly, the disease-associated vascular barrier dysfunction could be confirmed in human IBD patients indicating the clinical relevance of the findings. Imatinib (brand name Gleevec) is a kinase inhibitor acting against Abelson tyrosine kinase BCR–ABL, the KIT and PDGF receptors and is used for therapy of chronic myeloid leukemia (CML), gastrointestinal stromal tumors (GIST) and several other malignancies (88). Interestingly, treatment with imatinib restored adherens junctions, inhibited vascular permeability, and significantly reduced colonic inflammation in experimental colitis. Altogether, these results highlighted the pathogenic impact of inflammation–associated vascular barrier defects in IBD and opens new avenues for vascular-directed treatment of the disease (81).

The detection of an additional intestinal barrier rises the question whether the epithelial and the vascular barriers may communicate in prevention or progression of the disease. Epithelial to endothelial cell communication is commonly accepted. For example, the nutrient composition of the chyme (partially digested food) and not simply gut distension modulates blood flow. Specialized subsets of intestinal epithelial cells transport nutrients through the epithelial monolayer into the lamina propria from where they are transported through the fenestrated blood endothelium to be distributed systemically (89, 90). Moreover, in response to pathogen invasion or loss of barrier integrity, both intestinal epithelial cells and tissue-resident leukocytes secrete cytokines, chemokines, reactive oxygen species, and lipid mediators that activate endothelial cells to modulate the number and structure of vessels and to promote immune cell extravasation. For example, intestinal epithelial cells in IBD were shown to secret the chemokines CXCL8/IL-8 and CCL20 (91, 92), both of which can activate angiogenesis (93, 94). In addition, these cells secrete the cytokine TNF-α (91), which regulates vessel remodeling and by directly acting on endothelial cells may inhibit angiogenesis (95, 96). In addition, vascular permeability is increased by inflammatory mediators released from epithelial cells fostering both, inter- and trans-cellular diapedesis (90, 97).

## Angiocrine Functions of Blood Vessels in Organ Development and Diseases

The endothelium is not a passive response organ for nutrient supply, tissue entry of immune cells, and metabolite removal, but actively regulates the tissue microenvironment in organ development and diseases as indicated by novel results. These perfusion-independent functions of endothelial cells were recognized in experimental tumor models in mice for the first time, where the inhibition of angiogenesis in certain instances did not abrogate tumor growth but instead enhanced tumor invasiveness (98). Based on this the hypothesis arose that endothelial cells release specific soluble factors that may directly regulate tumor growth in a perfusion-independent manner. This respective mechanism was termed as “angiocrine” regulation of tumorigenesis (98).

Subsequent studies confirmed that endothelial cells may activate tumorigenesis by secreted factors (98, 99). For example, angiocrine factors were reported to stimulate growth and migration of lymphoma tumor cells (100), to maintain stem cell like properties in colorectal carcinoma and glioblastoma cells (101–103), to inhibit anoikis in head and neck cancer stem cells (104) and, to activate proliferation, survival and epithelial to mesenchymal transition of lung carcinoma cells (103) [for review see: Lee et al. (105)].

*Vice versa*, it was noted that endothelial cells can also suppress cancer growth through angiocrine signaling. In this framework contact-dependent interactions between the endothelial cell surface receptor duffy antigen/receptor for chemokines and the carcinoma cell surface receptor kang ai-1 were shown to suppress metastasis (106). In addition, in breast cancer endothelial cell-released slit homolog 2 protein (Slit 2), perlecan and additional as yet unknown factors were reported to inhibit proliferation, invasion and pro-tumorigenic signaling of the cancer cells (107, 108). In addition, thrombospondin is regarded as a putative anti-angiogenic factor secreted from endothelial cells (98). Angiocrine factors also exert key functions in physiologic condition such as kidney development (109, 110), liver bud (111) and pancreatic bud formation (112), in neuronal development (113), lung regeneration (114), osteogenesis (115) and hematopoiesis (113).

Of note, a specific impact of angiocrine signaling on epithelial barrier functions was observed in retina development (116). Endothelial cells secrete factors that remodel the retinal pigment epithelium (RPE) basement membrane and integrin receptors sense these changes by triggering GTPase signals that modulate RPE tight junctions and enhance RPE barrier function (116). Similar parenchymal cell barrier regulatory mechanisms may be active in other organs.

Altogether, angiocrine factors are involved in tumorigenic, homeostatic, regenerative and morphogenetic processes in a paracrine or juxtacrine manner. The term “angiocrine” factors meanwhile includes secreted and membrane-bound inhibitory or stimulatory growth factors, trophogens, chemokines, cytokines, extracellular matrix components, exosomes and other cellular products (117). The angiocrine profile of endothelial cells can differ between tissues, reflecting the diversity of cell types found adjacent to endothelial cells in organs (113, 117).

## The Impact of Angiocrine Signaling on Epithelial Barrier Function in IBD

Angiocrine functions in IBD have not been investigated extensively as yet, despite the manifold effects of angiocrine signaling on epithelial cell functions in cancer, organ development and tissue regeneration. However, first results indicating angiocrine activities in the colon have emerged. For example, endothelial cells release jagged 1, generated by proteolytic activity of ADAM metallopeptidase domain 17 (ADAM17) activating Notch in human colorectal cancer cells and thereby promoting a cancer stem cell phenotype and chemo-resistance (103, 118). Moreover, it was shown that selectively endothelial cells isolated from colorectal carcinomas with a prognostically favorable Th-1-like immune environment released the matricellular protein *secreted protein, acidic and rich in cysteine–like 1 (SPARCL1)*, which autocrinely and paracrinely inhibited angiogenesis and proliferation of different cancer cell lines (119, 120). The latter indicated that angiocrine activities in the colon may trigger the course of diseases in a microenvironment–dependent manner. A recent single cell RNAseq approach of intestinal cells and subsequent bioinformatics interaction analyses supported the molecular interaction between endothelial cells and epithelial cells in the colon (121).

Specific support for angiocrine functions in IBD was obtained from a recent report on an increased susceptibility for acute and chronic DSS-induced colonic inflammation in mice lacking the angiocrinely active SPARCL1 protein (122). SPARCL1 is almost exclusively expressed in vascular cells in the colon (119, 123, 124). In SPARCL1 (Sc1) KO animals colonic inflammation and colon vessel permeability were significantly increased and colon length was shorter as compared to wildtype animals. Exaggerated inflammation in Sc1 KO animals was further supported by an increased detection of fibrosis and the presence of tertiary lymphoid structures similar to the human chronic disease. Altogether, these results indicated that intestinal angiocrine functions may establish a chemical barrier affecting both, epithelial and endothelial cell barrier functions in IBD (122).

In a next step, we applied a meta-analysis to further investigate whether angiocrine signaling may impact barrier functions. To this goal, an *in silico* secretome screening against the human proteome was performed using the VerSeDa database [Vertebrate Secretome Database (125)]. Transcripts with a prediction cut-off value > 0.8 (SignalP 4.1, TargetP 1.1, SecretomeP) were considered as secreted proteins. The resulting 1,050 genes (1,959 proteins; 1,959 gene transcripts) were used for a functional gene and phenotype annotation using the Ensembl BioMart database (http://www.ensembl.org/index.html). Next, candidates were selected based on data mining (inflammatory, angiocrine, epithel, extracellular, endothelial, barrier, cytokine, bowel, secreted). Subsequently, the resulting 257 genes were mapped to profiles from human endothelial cells of different origin, including human umbilical vein endothelial cells (HUVEC) exposed to shear stress (126), under LPS-stimulation (127), overexpressing γ-interferon-inducible protein (IFI) 16 (128) and unstimulated (129), as well as endothelial cells from brain, lung, heart (130) and colorectal carcinoma (119, 131). This analysis identified in total 28 genes (Table 1). Six of these may be of specific interest as candidates of angiocrine barrier effects in IBD (Figure 1B). This includes components of the von Willebrand factor domain superfamily (VWA1, VWF) and tissue inhibitor of metalloproteinases (TIMP)-1, which were retrieved from three different studies, respectively. vWF is a classical endothelial cell marker protein, that promotes adhesion of platelets to the sites of vascular injury by forming a molecular bridge between sub-endothelial collagen matrix and the platelet-surface receptor complex (132). Its impact on the epithelial barrier warrants further investigation. TIMP-1 is an inhibitor of the matrix metalloproteinases (MMPs). It is able to promote cell proliferation in a wide range of cell types, has an anti-apoptotic function and can modulate the vascular barrier (133, 134). TIMP-1 may impact the epithelial cell barrier activity in the gut through these activities. In this framework, it is interesting that MMP-14 was also identified by our meta-analyses as angiocrine mediator. MMP-14 was reported as an angiocrine factor in lung regeneration and as a member of the membrane-type matrix metalloproteinases that are not inhibited by TIMP-1 (114, 135). In addition, CXCL10, regarded as a major driver in IBD pathogenesis (6), was also identified as angiocrine mediator in our meta analyses. In the DSS-model blockade of CXCL10 enhanced crypt cell survival (136) and mice with a knock out of the CXCL10 receptor CXCR3 showed considerably lower crypt damage (137). Based on these findings it was suggested that CXCL10 may exert direct effects on epithelial cells in the gut (138).

**Table 1:** Angiocrine barrier-modulating candidate genes in inflammatory bowel disease.

**Gene**	**Full name** **(according to GeneBank)**	**Alias** **(GeneBank)**	**GeneID** **(GeneBank)**	**Burghoff** **et al. (126)**	**Tunica** **et al. (129)**	**Kwon** **et al. (127)**	**Jambusaria** **et al. (130)**	**Baggetta** **et al. (128)**	**Naschberger** **et al. (119)**
CLU	Clusterin	AAG4, APO-J, APOJ, CLI, CLU1, CLU2, KUB1, NA1/NA2, SGP-2, SGP2, SP-40, TRPM-2, TRPM2	1191	x					
CST3	Cystatin C	ARMD11, HEL-S-2	1471	x					
FBN2	Fibrillin 2	CCA, DA9, EOMD	2201	x					
GDF15	Growth differentiation factor 15	GDF-15, MIC-1, MIC1, NAG-1, PDF, PLAB, PTGFB	9518	x					
MGP	Matrix Gla protein	GIG36, MGLAP, NTI	4256	x					x
EDN1	Endothelin 1	ARCND3, ET1, HDLCQ7, PPET1, QME	1906	x					
IGF2	Insulin like growth factor 2	C11orf43, GRDF, IGF-II, PP9974, SRS3	3481	x			x		
TIMP1	TIMP metallopeptidase inhibitor 1	CLGI, EPA, EPO, HCI, TIMP, TIMP-1	7076	x	x		x		
LOXL2	Lysyl oxidase like 2	LOR, LOR2, WS9-14	4017	x	x				
CST1	Cystatin SN	-	1469			x			
A2M	Alpha-2-macroglobulin	A2MD, CPAMD5, FWP007, S863-7	2			x			
MMP14	Matrix metallopeptidase 14	MMP-14, MMP-X1, MT-MMP, MT-MMP 1, MT1-MMP, MT1MMP, MTMMP1, WNCHRS	4323		x				
FBLN1	Fibulin 1	FBLN, FIBL1	2192		x				
VWF	Von Willebrand factor	F8VWF, VWD	7450		x		x		
PDIA3	Protein disulfide isomerase family A member 3	ER60, ERp57, ERp60, ERp61, GRP57, GRP58, HEL-S-269, HEL-S-93n, HsT17083, P58, PI-PLC	2923		x				
WFDC2	WAP four-disulfide core domain 2	EDDM4, HE4, WAP5, dJ461P17.6	10406				x		
BSG	Basigin (Ok blood group)	5F7, CD147, EMMPRIN, EMPRIN, HAb18G, OK, SLC7A11, TCSF	682				x		
CXCL10	C-X-C motif chemokine ligand 10	C7, IFI10, INP10, IP-10, SCYB10, crg-2, gIP-10, mob-1	3627				x		
PTGDS	Prostaglandin D2 synthase	L-PGDS, LPGDS, PDS, PGD2, PGDS, PGDS2	5730				x		
SAA2	Serum amyloid A2	SAA, SAA1	6289				x		
SAA1	Serum amyloid A1	PIG4, SAA, SAA2, TP53I4	6288				x		
ICAM1	Intercellular adhesion molecule 1	BB2, CD54, P3.58	3383				x	x	
SPARCL1	SPARC like 1	MAST 9, MAST9, PIG33, SC1, hevin	8404				x		x
VWA1	Von Willebrand factor A domain containing 1	WARP	64856				x		x
FGFR1	Fibroblast growth factor receptor 1	BFGFR, CD331, CEK, ECCL, FGFBR, FGFR-1, FLG, FLT-2, FLT2, HBGFR, HH2, HRTFDS, KAL2, N-SAM, OGD, bFGF-R-1	2260						x
PTGS1	Prostaglandin-endoperoxide synthase 1	COX1, COX3, PCOX1, PES-1, PGG/HS, PGHS-1, PGHS1, PHS1, PTGHS	5742						x
CTSH	Cathepsin H	ACC-4, ACC-5, ACC4, ACC5, CPSB	1512						x
TNFRSF1B	TNF receptor superfamily member 1B	CD120b, TBPII, TNF-R-II, TNF-R75, TNFBR, TNFR1B, TNFR2, TNFR80, p75, p75TNFR	7133						x

The bioinformatical analysis showed that the overlap of genes retrieved from the different studies was low. This is well in agreement with the high variation of activation and organ-dependent plasticity of endothelial cells. In this framework, the six genes identified in endothelial cells from colorectal carcinoma may exhibit the highest relevance for IBD (see Table 1). Interestingly, SPARCL1, which has been shown to affect susceptibility to experimental colitis in mice was part of this group (122). In summary, this analysis identified several interesting candidates, which may participate in the angiocrine inter-barrier communication in IBD. These factors may provide putative new targets for treatment of the disease. The specific impact of most of these factors on the epithelial barrier functions has to be determined in future studies.

## Conclusion

First evidence exists that the gut-vascular barrier (GVB) communicates via angiocrine signals with the epithelial barrier during IBD. The molecules involved in this communication may provide new targets for clinical monitoring and treatment of the disease. In-depth elucidation of the underlying effects and the specific mechanisms warrants further studies.

## Author Contributions

MS, SK, and EN: analyzed the literature and wrote the manuscript. MK performed the bioinformatical analysis. All authors approved the final version of the manuscript.

## Conflict of Interest

The authors declare that the research was conducted in the absence of any commercial or financial relationships that could be construed as a potential conflict of interest.

## Publisher's Note

All claims expressed in this article are solely those of the authors and do not necessarily represent those of their affiliated organizations, or those of the publisher, the editors and the reviewers. Any product that may be evaluated in this article, or claim that may be made by its manufacturer, is not guaranteed or endorsed by the publisher.
